# [18F]-FDG PET-CT in Malignant Melanoma

**DOI:** 10.3390/diagnostics15101192

**Published:** 2025-05-08

**Authors:** Teodora Sidonia Mititelu, Mihaela Raluca Mititelu, Sandica Bucurica, Daniel Octavian Costache

**Affiliations:** 1 Department of Plastic and Reconstructive Surgery, “Bagdasar-Arseni” Clinical Emergency Hospital, 041915 Bucharest, Romania; teodora-sidonia.mititelu0125@rez.umfcd.ro; 2 Department of Nuclear Medicine, Carol Davila University of Medicine and Pharmacy, 020021 Bucharest, Romania; 3 Clinic of Nuclear Medicine, University Emergency Central Military Hospital “Dr. Carol Davila”, 024185 Bucharest, Romania; 4 Department of Internal Medicine and Gastroenterology, Carol Davila University of Medicine and Pharmacy, 020021 Bucharest, Romania; 5 Clinic of Gastroenterology, University Emergency Central Military Hospital “Dr. Carol Davila”, 024185 Bucharest, Romania; 6 Department of Dermatology, Carol Davila University of Medicine and Pharmacy, 020021 Bucharest, Romania; daniel.costache@umfcd.ro; 7 Clinic of Dermatology, University Emergency Central Military Hospital “Dr. Carol Davila”, 024185 Bucharest, Romania

**Keywords:** malignant melanoma, PET-CT, [18F]-FDG, metabolic parameters, Breslow thickness, ulceration, tumor-infiltrating lymphocytes, metastasis

## Abstract

**Background/Objectives**: Malignant melanoma (MM) is an aggressive neoplasm with a rising global incidence. Accurate staging and risk stratification are essential for guiding therapeutic decisions and improving patient prognosis. [18F]-FDG PET-CT enables the non-invasive assessment of tumor metabolic activity, offering a valuable adjunct to histopathological evaluation. However, the correlation between PET-CT findings and established prognostic markers in MM, such as Breslow thickness, ulceration, and mitotic rate, remains insufficiently explored. **Methods**: This retrospective observational study included 61 patients diagnosed with MM, of whom 48 met the inclusion criteria. Quantitative and qualitative variables such as SULmax, Breslow thickness, Ki-67 expression, and mitotic rate were analyzed using descriptive statistics, while correlations between PET-CT findings, SLNB, and histopathological characteristics were assessed using Spearman’s correlation test. A *p*-value < 0.05 was considered statistically significant. **Results**: Significant associations were identified between ulceration and both overall metastases (*p* = 0.01) and pulmonary metastases (*p* = 0.02). Breslow thickness showed a positive correlation with metastatic spread (*p* = 0.01), reinforcing its role as a key prognostic indicator. Perineural and vascular invasion were significantly associated with intra-abdominal metastases (*p* < 0.001 and *p* = 0.0007, respectively). Tumor-infiltrating lymphocytes (TILs) were inversely correlated with intra-abdominal metastases (*p* = 0.05), while sentinel lymph node positivity correlated with the presence of regional (*p* = 0.008) and distant (*p* = 0.02) metastases. Additionally, subcutaneous SULmax values were significantly higher in male patients compared to females (*p* = 0.04). **Conclusions**: Integrating PET-CT metabolic parameters with histopathological markers enhances the assessment of MM aggressiveness and metastatic potential. By refining risk stratification, PET-CT may contribute to personalized therapeutic strategies and improved patient management in MM.

## 1. Introduction

Malignant melanoma (MM) is one of the most aggressive cutaneous neoplasms, with a globally increasing incidence. According to GLOBOCAN 2022, over 300,000 new cases are reported annually, emphasizing the need for early diagnosis and treatment [1]. The prognosis of MM patients depends on accurate staging, which remains essential for guiding therapeutic decisions and improving survival rates [2,3].

The use of hybrid imaging, particularly PET-CT with radiolabeled glucose analog ([18F]-FDG), is a major area of interest in current melanoma management. This imaging technique allows for non-invasive assessment of tumors’ metabolic and structural characteristics and facilitates the early detection of distant lesions, which may not be visible through conventional anatomical imaging techniques such as ultrasound, CT, and MRI [4]. This approach has the potential to complement and expand the information obtained through clinical examination and histopathological analysis. However, the relationship between hybrid imaging findings and established markers of melanoma aggressiveness—such as Breslow thickness, mitotic rate, and the presence of ulceration—requires a more detailed and rigorous analysis. This represents the key focus of the study, which aims to clarify how imaging can contribute to a better understanding of the nature and behavior of MM.

Recognized for its rapid progression and metastatic potential, malignant melanoma requires a precise assessment of tumor aggressiveness to ensure optimal management. This study aims to integrate hybrid imaging with clinical and histopathological data to improve the risk stratification and staging of MM.

This study seeks to integrate the perspectives offered by hybrid FDG PET-CT imaging with clinical and histopathological parameters (including Breslow thickness, mitotic rate, ulceration presence, and sentinel lymph node status), as well as with demographic data related to MM pathology, in order to characterize tumor aggressiveness. This approach has the potential to contribute to the optimization of personalized therapeutic strategies and the improvement of patient prognosis. Furthermore, by systematically integrating hybrid imaging biomarkers with pathological indicators of tumor aggressiveness, the present study could serve as a basis for future applications of radiomics in melanoma, supporting the development of advanced non-invasive predictive models.

## 2. Materials and Methods

### 2.1. Study Design

This retrospective observational study included consecutive patients with confirmed diagnosis of MM, referred for PET-CT evaluation to the Clinic of Nuclear Medicine at the Dr. Carol Davila University Emergency Central Military Hospital Bucharest between 10 January 2022 and 26 September 2023. Imaging and histopathological data were retrieved from the records of the Clinic of Nuclear Medicine.

### 2.2. Patient Selection

The initial cohort consisted of 61 adult patients, of whom 48 met the inclusion criteria, defined as follows: (1) histopathologically confirmed malignant melanoma diagnosis, (2) availability of PET-CT investigation results, and (3) availability of histopathologic data. From this cohort, 13 patients were excluded based on the following criteria: (1) presence of concomitant neoplasm or history of malignancy in the past 10 years, (2) missing documentation required for study participation, and (3) inconclusive PET-CT examination due to non-compliance with pre-scan fasting instructions.

### 2.3. PET-CT

Prior to the examination, all patients adhered to a 6 h fasting protocol, ensuring blood glucose levels did not exceed 200 mg/dL. Additionally, patients were advised to avoid physical exertion on the day before the scan to prevent FDG uptake in muscle tissue and minimize imaging artifacts [5]. [18F]FDG was administered intravenously via a peripheral catheter at a dose of 3 MBq/kg, using a computerized dose fractionation and delivery system. Image acquisition was performed 60 min after radiopharmaceutical administration. The examination was conducted using the Discovery MI-DR system (GE Healthcare, Chicago, IL, USA), employing a 64-slice CT scanner and a standardized melanoma imaging protocol (vertex-to-plantar scan, including both upper and lower limbs).

### 2.4. Standardized Uptake Value (SUV)

SUV (standardized uptake value) is a measure of the relative absorption of the radiopharmaceutical within a region of interest (ROI). Tumor activity is usually expressed using SUVmax, which represents the highest pixel intensity within the ROI. This approach helps to exclude low-value areas caused by necrosis or adjacent normal structures [6]. In this study, all SUV values were calculated using LBM correction (lean body mass—SUL, SULmax), making it a more precise metric for individuals with a high percentage of body fat [7]. Additionally, SULmean, which calculates the mean of all SULmax values in the region of interest, is considered more representative, as it helps to reduce the likelihood of false positive results [8].

### 2.5. Histopathology and Immunohistochemistry

In this study, key variables were analyzed to assess tumor aggressiveness and its correlation with PET-CT imaging findings. Clinical subtypes of melanoma, ulceration, Breslow thickness, mitotic rate, tumor-infiltrating lymphocytes (TILs), and Ki-67 expression were evaluated. These parameters were correlated with PET-CT findings to explore their relationship with tumor metabolism and progression.

### 2.6. Clinical Variants of Malignant Melanoma

The clinical variants of MM reflect the diversity of cutaneous neoplasia, with each presenting distinct characteristics regarding manifestation, progression, and response to treatment. Although not included in the 8th AJCC classification, precise identification and classification of these variants is essential for optimizing clinical management and assessing disease progression [3].

Thus, invasive MM is classified, based on clinical and histopathological characteristics, into four major histological subtypes (Table 1).

### 2.7. Ulceration

Ulceration is defined as the loss of the epidermal matrix [10]. This qualitative variable is considered in this study for serial comparison with both other histopathological markers and quantitative PET-CT parameters.

### 2.8. Breslow Thickness

Breslow thickness measures the maximum depth of tumor invasion from the granular layer (or, in ulcerated tumors, from the base of the ulceration) to the deepest point of invasion. This index is a key prognostic indicator and plays a crucial role in guiding therapeutic strategies for MM patients, as outlined in the 8th AJCC classification [3]. In addition to serving as a descriptive characteristic for the study cohort, this quantitative variable was also analyzed in relation to the other examined parameters.

### 2.9. Mitotic Rate

The mitotic rate in MM represents the number of cell divisions (mitoses) observed per unit area (typically per square millimeter) within the invasive portion of the tumor. In previous editions of the AJCC classification criteria, the mitotic rate was included in the T-category staging criteria for MM. However, in the latest edition, it was removed from the TNM staging criteria. Nevertheless, it remains an important prognostic factor, with a significant association with melanoma-specific survival [3].

### 2.10. Tumor-Infiltrating Lymphocytes (TILs)

The tumor microenvironment is a complex entity and a subject of interest in the era of immunotherapies. In addition to tumor cells, it includes a wide range of immune cells, among which TILs are recognized for their strong responsiveness to the presence of the tumor component. These include T and B lymphocytes, as well as natural killer (NK) cells, with each subgroup exhibiting distinct activity patterns [11]. Histopathological analysis of lymphocytic infiltration patterns distinguishes three classifications: brisk (reflecting a uniform and extensive lymphocyte presence), non-brisk (meaning focal lymphocytic infiltration), and absent (indicating a complete lack of lymphocytic infiltration). These reflect the degree and intensity of TILs’ presence within the tumor tissue [12,13].

### 2.11. Ki-67

Ki-67 is a nuclear protein associated with cell proliferation and is widely used as an oncology marker for assessing tumor cell division rates, providing insight into tumor aggressiveness. Thus, a high Ki-67 level indicates a high proliferation rate, suggesting more aggressive tumor behavior with an increased risk of metastasis and recurrence. In both clinical practice and research, Ki-67 is often used alongside other markers to provide a more comprehensive tumor profile. While its prognostic role remains a subject of debate, Ki-67 was included in this study as a quantitative variable in order to correlate tumor aggressiveness with paraclinical data at presentation.

### 2.12. Sentinel Lymph Node Biopsy (SNLB)

The sentinel lymph node (SLN) is the first to receive lymphatic drainage from the primary tumor, serving as an early indicator of nodal metastases and disease progression [14,15]. SLNB is used in clinically node-negative MM patients to detect microscopic metastases for tumors with >1 mm Breslow thickness, or <1 mm with high-risk features (ulceration, high mitotic rate, regression ≥50%) [3,16]. Histopathological evaluation included H&E staining and immunohistochemistry.

Of the 48 patients included in the study, 30 underwent SLNB to assess microscopic nodal metastases. SLNB results were analyzed in correlation with histopathological markers and PET-CT findings to refine staging and prognosis.

### 2.13. Statistical Analysis

Data analysis was performed using SPSS (version 29.0), EpiInfo (version 7.1.5), and Microsoft Office Excel 2019, as follows: Qualitative variables (e.g., sex, tumor location, clinical variant, key histopathological features, and presence or absence of metastases as determined by PET-CT) were analyzed by calculating frequency distributions. Quantitative variables with normal distribution (e.g., age, primary tumor thickness, Ki-67 expression, mitotic count, and SULmax) were analyzed using the median to characterize central tendency and the shape of the distributions (e.g., age, primary tumor thickness, Ki-67 expression, mitotic count, and SULmax). Additionally, Spearman’s correlation test was used to assess the relationships between the key histopathological features, such as SLNB positivity, and the presence/location of metastases. The Mann–Whitney U test was applied to evaluate the correlation between patient sex and SULmax values at metastatic sites. A *p*-value < 0.05 was considered statistically significant.

## 3. Results

### 3.1. Demographic Data and Basic Tumor Characteristics

Among the 61 patients examined, a total of 48 patients (78.69%) met the inclusion criteria; of these, 31 were women and 17 were men.

The mean age at the time of PET-CT investigation was 61 years. It is important to note a standard deviation of approximately 14 years, reflecting a bimodal age distribution. Specifically, there were two predominant incidence peaks: one in the fifth decade (50–59 years) and another in the seventh decade (70–79 years). When analyzing the age distribution by sex, the highest incidence of malignant melanoma in men was in the 70–79 age group, whereas for women, the peak incidence was in the 50–59 age group.

Regarding the anatomical sites of primary tumor location, the chest was the most frequent site, followed by the lower limbs. Notably, melanoma of the nasal vestibule was rare, correlating with the low incidence of mucosal melanoma (Table 2).

In terms of clinical melanoma subtypes, the most frequently observed form was nodular melanoma (NM, 48%), followed by superficial spreading melanoma (SSM, 27%) and lentigo maligna melanoma (LMM, 13%). The acral lentiginous melanoma (ALM), mucosal melanoma, and Spitz melanoma variants were rare (<5%). There was also one case (2%) where the primary tumor could not be identified.

The median Breslow thickness and mitotic rate were 2.00 mm and 3 mitoses/mm^2^, respectively, reflecting moderate tumor invasiveness within the study cohort. Among the metabolic parameters, pulmonary and subcutaneous metastases demonstrated notable SULmax values, with medians of 4.90 and 3.86, respectively. The highest median SULmax was observed in bone metastases (9.05), underscoring their elevated metabolic activity. Missing data rates varied across metastatic sites, primarily due to the absence of corresponding lesions in many patients—this is the case for brain lesions which, usually, are not visible in [18F]-FDG PET-CT images due to the high physiological glucose uptake in normal brain tissue; however, a cerebral lesion was identified in one patient (Table 3).

**Figure 1:**
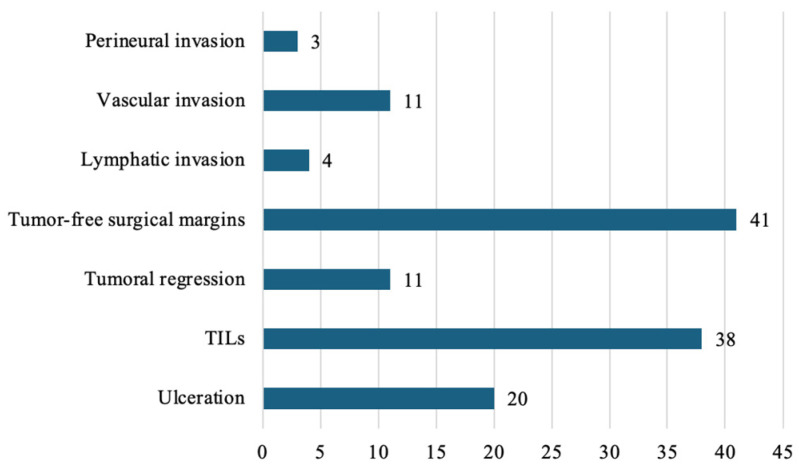
Distribution of key histopathological characteristics.

From a qualitative histopathological perspective, the majority of resected specimens had tumor-free surgical margins, indicating complete excision with a circumferential margin of healthy tissue—a positive prognostic factor in melanoma management. Conversely, tumor regression, which may suggest an immune response, and the presence of TILs, reflecting an immune response within the tumor microenvironment, were identified at a moderate frequency (Figure 1).

Features associated with tumor invasion, such as perineural, vascular, and lymphatic invasion, which are negative prognostic factors due to their increased potential for tumor dissemination, were observed in three, eleven, and four cases, respectively. Ulceration was present in 20 cases, suggesting the aggressiveness of tumors within the examined cohort (Figure 1).

Additionally, among the 48 patients included in the study, 30 underwent SLNB. Of these, 17 patients showed secondary dissemination. This indicates that 56.7% of patients who underwent SLNB had lymphatic melanoma spread, highlighting a relatively high incidence of metastases in the analyzed cohort. These findings integrate into the discussion of the role of hybrid imaging in MM assessment, emphasizing SLNB as a crucial tool for staging and identifying high-risk metastatic patients.

When analyzing metastasis distribution among MM-diagnosed patients, based on PET-CT findings, 77% of patients were diagnosed with metastases. The remaining 23% of patients showed no PET-CT evidence of metastases. Among patients with metastases, the mean number of detected secondary lesions per patient ranged between 1 and 17 (mean 8).

**Figure 2:**
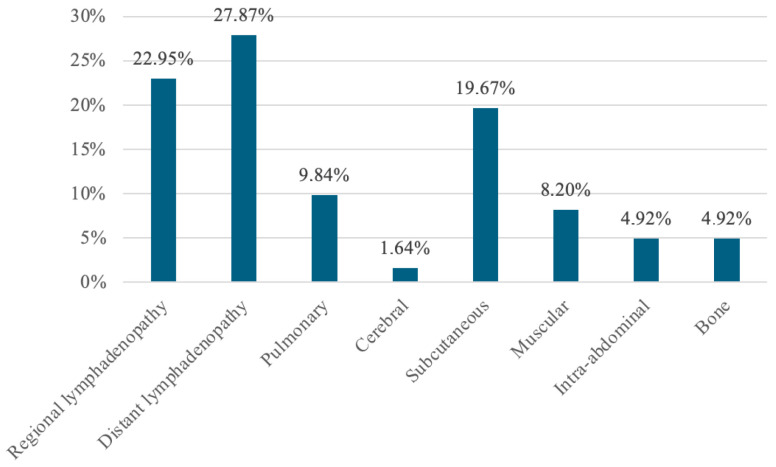
Distribution of metastases detected by PET/CT.

Regarding the distribution of secondary lesions, the most common metastatic sites were lymph nodes, present in 50.8% of cases, confirming melanoma’s tendency towards lymphatic dissemination. Specifically, regional lymph node metastases accounted for 22.95%, while distant lymphadenopathy was reported in 27.87% of cases. Bone, pulmonary, and muscular metastases were also recorded, with estimated prevalences of 4.92%, 9.84%, and 8.20%, respectively (Figure 2).

Despite PET-CT’s limitations in detecting all metastatic sites, particularly in tissues with high background metabolic activity, cerebral metastases were identified in one patient. It is important to note that only current PET-CT results were included, and no prior imaging examinations were analyzed.

### 3.2. Correlations Between Tumor Characteristics and PET-CT Findings

This study explores the relationship between the histopathological characteristics of malignant melanoma (MM), the detection of metastases through PET-CT, and sentinel lymph node (SN) positivity, using Spearman’s correlation coefficient, in order to identify statistical associations (Table 4).

Breslow thickness demonstrated a significant association with the presence of metastases (CC = 0.025, *p* = 0.01), suggesting that tumor thickness may serve as a predictor of metastatic spread. Additionally, a positive correlation was observed between ulceration and the presence of metastases (CC = 0.360, *p* = 0.01), emphasizing its role as a negative prognostic factor in MM. Furthermore, ulceration correlated specifically with the presence of pulmonary metastases (CC = 0.2319, *p* = 0.02), reinforcing its significance not only as a marker of local disease severity, but also as an indicator of pulmonary metastatic potential.

A strong correlation was identified between perineural invasion and intra-abdominal metastases (CC = 0.644, *p* < 0.001), as well as between vascular invasion and intra-abdominal metastases (CC = 0.573, *p* = 0.0007). This suggests that these types of invasion may play a role in predicting visceral tumor dissemination. Additionally, the histopathological presence of tumor-infiltrating lymphocytes (TILs) negatively correlated with intra-abdominal metastases (CC = −0.276, *p* = 0.05), indicating that a strong TIL presence in the tumor microenvironment may be associated with a lower incidence of intra-abdominal metastases, possibly due to an effective local immune response.

Interestingly, tumor regression was significantly negatively correlated with regional metastases (*p* = 0.01), potentially indicating an effective immune response against the primary tumor. Sentinel lymph node positivity showed a significant correlation with both regional lymph node metastases (*p* = 0.008) and the overall presence of metastases (*p* = 0.02), reaffirming the prognostic importance of SLNB in assessing regional metastatic spread. This finding reinforces the role of SLNB in identifying patients at high risk of regional lymphatic dissemination.

Additionally, Ki-67 showed a positive correlation with distant lymph node metastases (CC = 0.28, *p* = 0.05). This suggests that a higher proliferation rate, reflected by increased Ki-67 expression, may contribute to the metastatic capacity of MM in lymph nodes.

Another notable finding was the significant correlation between perineural invasion and bone metastases (CC = 0.289, *p* = 0.004), suggesting that the presence of perineural invasion may indicate an increased risk of bone metastases. This highlights the possibility that perineural invasion may serve as an early marker of skeletal tumor dissemination. Furthermore, a negative correlation was observed between the presence of bone metastases and Ki-67 proliferation index scores (CC = –0.30, *p* = 0.039), implying that tumors with lower proliferative activity may still exhibit a predilection for bone metastases.

### 3.3. Correlation Between SULmax Values and Patient Sex

The associations between SULmax values and patient sex across different anatomical regions were also analyzed (Table 5). Statistical analysis revealed differences in standardized uptake values (SULmax) between males and females with MM.

Notably, a significant difference was observed in the subcutaneous SULmax values (*p* = 0.04), with higher median values in males. Furthermore, the pulmonary SULmax values approached statistical significance (*p* = 0.057), suggesting a potential sex-related variation in metabolic activity at these metastatic sites.

## 4. Discussion

In this study, we explored significant correlations between histopathological characteristics, patient demographic data, and PET-CT findings. A significant association (*p* = 0.01) was observed between tumor thickness and the presence of radiotracer-avid lesions in PET-CT images. This correlation suggests that tumor thickness could serve as a strong predictor of metastatic spread, confirming our initial hypothesis. Tumor thickness, as an indicator of tumor invasiveness, is recognized as a key prognostic factor in MM [2]. The association between the Breslow index score and metabolic activity highlights the importance of early and precise measurement of this index, given its impact on secondary dissemination and patient survival. A recent study conducted on patients with acral lentiginous melanoma (ALM) by Xiaoting et al. reported that survival rates differ significantly for ALM patients in stages T1–T3, but not for those between T3 and T4. In other words, for tumors thicker than 2 mm, an increased Breslow index score does not necessarily correlate with a worse prognosis [17]. While our study confirmed a positive correlation between tumor thickness and metabolic activity, Xiaoting et al.’s findings suggest that for ALM, this relationship does not necessarily extend to long-term survival outcomes. This highlights the need for a more nuanced approach in melanoma prognosis evaluation, incorporating histopathological factors, PET-CT imaging data, and clinical variables such as the tumor subtype.

Ulceration is a significant predictor of tumor aggressiveness and was expected to correlate with higher metabolic activity in PET-CT-detected lesions, indicating increased metastatic potential in ulcerated MM. The results confirmed a positive correlation (*p* = 0.01) between ulceration and overall metastasis presence, emphasizing ulceration’s status as a negative prognostic factor in MM. According to Bønnelykke-Behrndtz et al., ulceration is recognized as a negative prognostic marker in primary cutaneous MM, being associated with decreased survival and shorter disease-free intervals [10]. This study also underscores ulceration’s role as a predictive marker for responses to immunotherapy. Additionally, Portelli et al. investigated whether the extent of ulceration predicts recurrence-free survival (RFS) and overall survival (OS) in primary cutaneous MM [18]. Their results demonstrated a significant interaction between Breslow index scores and ulceration extent, showing a negative impact of ulceration on both RFS and OS in patients with tumors ≤2 mm thick. Although the present study did not focus on interactions between different histopathological characteristics, a positive correlation between ulceration and pulmonary metastases (*p* = 0.02) was observed. This suggests that ulceration may serve not only as a marker of local disease severity, but also as an indicator of pulmonary metastatic potential. In the literature search conducted, no study was identified that establishes a direct correlation between MM ulceration and the presence of pulmonary metastases. However, Stadelmann et al. evaluated pulmonary nodules in AJCC stage III and IV MM patients, reporting that 68% of the analyzed nodules were metastatic, with at least one metastasis found in 78% of patients [19].

Furthermore, the analysis explored whether specific histopathological features, such as perineural invasion, vascular invasion, and tumor regression, are significant indicators of metastatic potential. Understanding how these markers influence prognosis and treatment strategies is crucial for MM patients. Our study demonstrated a strong correlation between perineural and vascular invasion and the development of intra-abdominal metastases. The significant positive correlation (*p* < 0.001 for perineural invasion and *p* = 0.0007 for vascular invasion) suggests that these invasion patterns may serve as important predictors of visceral tumor dissemination. This supports the hypothesis that perineural and vascular pathways facilitate distant melanoma spread. A study by Liebig et al. emphasized perineural invasion as an underestimated metastatic pathway [20]. It is a well-documented pathological characteristic in malignancies such as pancreatic, colorectal, and prostate cancer, where perineural invasion serves as a key prognostic marker. While Liebig’s study did not specifically address MM or intra-abdominal metastases, it reinforces perineural invasion as a distinct route of tumor dissemination, extending beyond local invasion to systemic spread.

Regarding vascular invasion as a prognostic factor in MM, Kashani-Sabet et al. demonstrated that vascular invasion is a critical indicator of prognosis in primary cutaneous MM [21]. The study described two mechanisms for detecting circulating malignant cells—either directly in the bloodstream or via proximity to the endothelium. The increased recurrence risk and reduced survival rates associated with vascular invasion underscore its importance as a prognostic factor comparable to ulceration.

Additionally, our study identified a correlation between perineural invasion and bone metastases (CC = 0.289, *p* = 0.04), suggesting a relevant association between these two phenomena. While no direct studies confirm an association between perineural invasion and MM bone metastases, both variables are well-established indicators of poor prognosis. A retrospective study by F. Tas reported that bone metastases are the fourth most common site of secondary MM dissemination [22]. MM preferentially metastasizes to the lungs, liver, and brain, with current therapies being strictly palliative, focusing on symptomatic management of secondary lesions [23].

Furthermore, Chang et al. highlighted the fact that perineural MM spread, though less common than that of squamous cell carcinoma, is a significant complication, particularly in desmoplastic head and neck MM [24]. The imaging features of perineural spread include abnormal thickening and contrast enhancement in MRI images, emphasizing the importance of careful radiological evaluation.

Our study also demonstrated a negative correlation between TIL presence and intra-abdominal metastases (CC = −0.276, *p* = 0.05). This suggests that the absence of TILs in the tumor microenvironment may be associated with a higher incidence of intra-abdominal metastases, particularly with hepatic involvement, leading to decreased survival. A possible explanation is an ineffective local immune response in these regions—a hypothesis supported by Conway et al., who highlighted the role of TILs in tumor progression suppression and improved prognosis in MM [25]. Their study found that higher TIL density was associated with better survival outcomes, reinforcing the concept that lymphocytic infiltration effectively counteracts metastasis. Notably, TIL density is lower in hepatic and cerebral metastases compared to pulmonary or subcutaneous metastases, which are associated with better survival rates [13].

The correlation between tumor regression and metastasis in MM is an area of ongoing interest. Our findings revealed a significant negative correlation between tumor regression and MM metastases (CC = −0.292, *p* = 0.043). This suggests that tumor regression may reflect an effective immune response, reducing metastatic potential. A study by El Sharouni et al. in patients from the Netherlands and Australia found that regression was associated with improved survival, especially in early-stage MM and superficial spreading melanoma (SSM) [26]. Additionally, the study published by Aivazian et al. concluded that tumor regression is associated with lower sentinel lymph node positivity rates and improved survival [27].

Finally, we investigated differences in metabolic activity in metastatic lesions in relation to sex, particularly in subcutaneous and pulmonary sites, suggesting that distinct biological variations may influence MM progression between sexes. The analysis of SULmax values by sex revealed a statistically significant difference in subcutaneous lesions (*p* = 0.041), with male patients demonstrating higher metabolic activity compared to females. This finding aligns with previous studies that suggest a more aggressive disease phenotype in males [28]. While sex-specific MM management was not the focus of this study, our findings suggest that sex may play a role in MM pathogenesis.

In addition to evaluating lymphatic spread with metabolic imaging, it is important to recognize the diagnostic limitations of FDG PET-CT in nodal staging and to emphasize the established clinical role of SLNB. While [18F]FDG PET-CT offers valuable whole-body assessment of metabolic activity, it is important to note its intrinsic limitations when attempting to detect micrometastases within lymph nodes. Sentinel lymph node biopsy (SLNB) remains the gold standard for evaluating regional nodal involvement, as it provides superior sensitivity by allowing the histological and immunohistochemical identification of microscopic disease [14]. The relatively lower spatial resolution of PET-CT limits its ability to detect subclinical nodal metastases, particularly in early-stage disease. Therefore, PET-CT should be regarded as complementary to, rather than a replacement for, SLNB when assessing nodal involvement.

One other limitation of FDG PET-CT is its reduced sensitivity towards small or superficially located primary cutaneous lesions. Specifically, in cases where the primary lesion presents as mild skin thickening, attenuation-corrected images may fail to reveal FDG uptake. In such scenarios, reviewing non-attenuation-corrected (NAC) images can be beneficial, as they are less affected by partial volume effects and may improve lesion detectability. This approach has been shown to enhance the detection of subtle cutaneous lesions, particularly when the primary site is clinically suspected but not definitively visualized in standard PET-CT reconstructions [29].

Despite the valuable insights gained, this study has several limitations that should be considered. The relatively small sample size of patients meeting the inclusion criteria may affect the broader applicability of the findings. Additionally, the retrospective nature of the study introduces heterogeneity in patient profiles and lesion characteristics, potentially influencing the results. The presence of confounding variables, such as prior treatments or non-neoplastic comorbidities, could further impact the observed associations. Lastly, it is important to note that the identified correlations do not imply causality, and further prospective studies are needed to validate these findings. Thus, future studies should focus on a prospective approach with a larger patient cohort, incorporating therapeutic strategies, concomitant pathologies, preventive measures (or their absence), and the genetic factors involved in MM pathogenesis. This comprehensive approach could further enhance the understanding of MM progression and optimize patient outcomes.

## 5. Conclusions

[18F]-FDG PET-CT demonstrates remarkable sensitivity in detecting secondary lesions, allowing for a detailed correlation between imaging findings and histopathological variables. Significant associations were observed, with overall and pulmonary metastases showing a strong correlation with ulceration. Intra-abdominal metastases detected using PET-CT were notably associated with perineural invasion, vascular invasion, and the presence of tumor-infiltrating lymphocytes (TILs). Distant lymph node metastases exhibited a significant correlation with the immunohistochemical proliferation marker Ki-67, while regional lymph node metastases were negatively correlated with tumor regression and positively associated with sentinel lymph node positivity. Additionally, bone metastases demonstrated a negative correlation with Ki-67, but a positive correlation with perineural invasion. Notably, subcutaneous SULmax values were higher in male patients compared to females.

By integrating imaging and histopathological data, this study offers valuable insights into tumor aggressiveness and metastatic potential. This combined approach enhances the identification and refinement of prognostic factors, facilitating more personalized and efficient management of MM patients. A multidisciplinary approach incorporating these findings may significantly improve clinical outcomes and optimize therapeutic decisions in accordance with evidence-based medicine.

## Figures and Tables

**Table 1:** Classification of MM ^1^.

Subtype	Characteristics
Superficial spreading melanoma (SSM)	The most common subtype, characterized by pagetoid extension within the epidermis
Nodular melanoma (NM)	Distinguished by a vertical growth pattern, with deep tissue invasion
Lentigo maligna melanoma (LMM)	Typically located on chronically sun-exposed surfaces
Acral lentiginous melanoma (ALM)	Occurring primarily on the palms, soles, and subungual regions

^1^ Modified after [9].

**Table 2:** Distribution of primary melanoma sites.

Location	Distribution (Number)
Thorax	15
Lower Limb	14
Upper Limb	9
Abdomen	1
Retroauricular	1
Rhinosinusal	1
Scalp	1
Unidentified	1

**Table 3:** Distribution of histopathological and PET parameters.

Parameter	Median (25th; 75th Percentile)
Breslow Index Score	2 (1.23; 4.75)
Mitotic Count	3 (1.63; 7)
SULmax Regional Lymphadenopathy	3.54 (2.17; 5.42)
SULmax Distant Lymphadenopathy	5.59 (3.86; 8.47)
Ki67%	15 (5; 20)
SULmax Pulmonary	4.9 (2.35; 6.98)
SULmax Subcutaneous	3.86 (2.56; 6.84)
SULmax Abdominal	9.05 (3.96; 9,61)
SULmax Muscular	4.77 (3.56; 8.05)
SULmax Bone	5.17 (3.46; 11.24)

**Table 4:** Correlation of PET-CT-detected metastases with key histopathological characteristics of MM and sentinel node positivity. CC—Spearman’s correlation coefficient.

Variable		Metastases Presence	Pulmonary	Intra-Abdominal	Distant Lymph Nodes	Regional Lymph Nodes	Muscular	Bone	Subcutaneous
Breslow Index	CC	0.025	0.016	−0.009	0.122	0.267	0.128	−0.18	0.073
	*p*	0.01	0.22	0.35	0.40	0.67	0.386	0.22	0.62
Lymphatic Invasion	CC	−0.014	−0.114	−0.077	0.091	−0.026	0.103	0.233	0.174
	*p*	0.92	0.44	0.59	0.53	0.85	0.49	0.11	0.24
Vascular Invasion	CC	0.179	−0.056	0.473	−0.092	−0.241	−0.186	0.267	0.258
	*p*	0.22	0.70	0.0007	0.53	0.09	0.21	0.065	0.08
Perineural Invasion	CC	0.141	−0.098	0.644	−0.011	−0.166	0.08	0.289	0.480
	*p*	0.34	0.51	<0.001	0.94	0.26	0.55	0.04	0.11
Tumor Regression	CC	−0.292	−0.206	−0.141	0.114	−0.35	0.024	−0.13	−0.086
	*p*	0.043	0.160	0.34	0.44	0.01	0.87	0.38	0.56
Ulceration	CC	0.360	0.2319	−0.044	0.169	0.108	0.265	0.131	0
	*p*	0.01	0.02	0.77	0.25	0.46	0.07	0.38	1
Mitotic Rate	CC	0.115	0.124	−0.181	0.188	0.153	0.129	0.141	0.002
	*p*	0.43	0.40	0.22	0.19	0.30	0.38	0.33	0.99
Ki67	CC	0.098	0.27	−0.031	0.2799	0.015	−0.063	−0.30	0.240
	*p*	0.50	0.56	0.831	0.05	0.92	0.67	0.034	0.1
Tumor-Free Surgical Margins	CC	−0.085	0.156	0.107	−0.188	0.265	0.141	−0.14	−0.170
	*p*	0.566	0.29	0.47	0.20	0.06	0.34	0.35	0.24
TILs	CC	0.035	0.176	−0.276	−0.037	0.121	0.026	−0.05	0.011
	*p*	0.81	0.23	0.05	0.80	0.41	0.86	0.75	0.94
Positive SN	CC	0.404	0.343	−0.157	0.190	0.476	0.234	0.234	0.101
	*p*	0.02	0.06	0.41	0.314	0.008	0.21	0.21	0.59

**Table 5:** Association between SULmax values and patient sex.

Parameters	Sex	Median (25th; 75th Percentile)	*p*
SULmax Regional Lymph Nodes	Female	3.25 (2.17; 4.12)	0.78
	Male	4.38 (2.72; 4.67)	
SULmax Distant Lymph Nodes	Female	5.51 (3.6; 8.86)	0.88
	Male	5.89 (4.12; 6.56)	
SULmax Pulmonary	Female	6.05 (5.01; 8.51)	0.057
	Male	2.35 (1.34; 3.08)	
SULmax Subcutaneous	Female	2.60 (2.34; 3.50)	0.041
	Male	4.21 (3.96; 10.19)	

## Data Availability

Data are available upon reasonable request.
